# The Role of Chemokines in Cervical Cancers

**DOI:** 10.3390/medicina57111141

**Published:** 2021-10-21

**Authors:** Fabian Garrido, Carl Mathis Wild, Johanna Mittelberger, Franziska Dobler, Mariella Schneider, Nadine Ansorge, Melitta Köpke, Annamarie Strieder, Nina Ditsch, Udo Jeschke, Christian Dannecker

**Affiliations:** Department of Obstetrics and Gynecology, University Hospital Augsburg, Stenglinstrasse 2, 86156 Augsburg, Germany; fabian.garrido@uk-augsburg.de (F.G.); mathis.wild@uk-augsburg.de (C.M.W.); johanna.mittelberger@uk-augsburg.de (J.M.); Franziska.dobler@uk-augsburg.de (F.D.); Mariella.schneider@uk-augsburg.de (M.S.); nadine.ansorge@uk-augsburg.de (N.A.); melitta.koepke@uk-augsburg.de (M.K.); annamarie.strieder@uk-augsburg.de (A.S.); nina.ditsch@uk-augsburg.de (N.D.); Christian.dannecker@med.uni-augsburg.de (C.D.)

**Keywords:** CCL1-28, CXCL1-17, XCL1-2, CX3CL1, immune checkpoints, PD-1, PD-L1

## Abstract

Both clinical-pathological and experimental studies have shown that chemokines play a key role in activating the immune checkpoint modulator in cervical cancer progression and are associated with prognosis in tumor cell proliferation, invasion, angiogenesis, chemoresistance, and immunosuppression. Therefore, a clear understanding of chemokines and immune checkpoint modulators is essential for the treatment of this disease. This review discusses the origins and categories of chemokines and the mechanisms that are responsible for activating immune checkpoints in cervical dysplasia and cancer, chemokines as biomarkers, and therapy development that targets immune checkpoints in cervical cancer research.

## 1. Introduction

With around 565,000 new cases per year, cervical cancer (CC) is the second most frequent female cancer and the third leading cause for cancer death in female patients worldwide [1,2]. The two main malignant epithelial cervical cancer types are the squamous cell carcinoma and the adenocarcinoma (about 70% and 10–25% of all cervix carcinomas, respectively) [3]. A persistent infection with high-risk human papillomavirus (HR-HPV) is the major leading cause of cervical cancer [4]. The tumor micro-environment consists of tumor-infiltrating lymphocytes (TIL) and tumor-associated macrophages (TAM), leading to a Treg and M2 differentiated/polarized immune response [5].

Chemokines, a subgroup of cytokines, are defined as signaling molecules which are responsible for chemotaxis. According to their spacing of the two conserved N-terminal cysteines they are subdivided into four groups: CXC, CC, CX3C, and C.

The CC chemokine proteins (or *β* chemokine proteins) have two adjacent cysteine (amino acids) near their *N*-terminus. At least 27 different members of this subgroup have been described for mammals called CC chemokine ligands (CCL)-1 to -28; CCL10 is identical to CCL9 [6,7,8,9,10,11]. Members of the CXC chemokine subfamily, on the other hand, have an intermediate amino acid between the first two cysteine; members of the CC chemokine subfamily have two adjacent cysteine. Normally, and with only a few exceptions, members of CXC chemokines are chemotactic for neutrophils, and CC chemokines are chemotactic for monocytes and a small subset of lymphocytes [12,13,14,15,16,17]. Chemokine (C-motif) ligands (XCL1 and -2) are small cytokines from the family of C chemokines, also known as lymphotactin. In humans, XCL1 is closely related to XCL2, whose gene localization can also be found on chromosome 1. Both have many genetic and functional similarities; however, XCL2 has so far only been described in the human system. XCL1, together with its receptor XCR1, is involved in cross-presentation, antigen uptake, and the induction of congenital and adaptive cytotoxic immunity. XCR1 is expressed exclusively in conventional dendritic cells. XCL1 is secreted by antigen-specific CD8 + T cells and NK cells along with a number of other chemokines. CX3CL1, also known as fractalkine, triggers its adhesive and migration functions by interacting with the CX3CR1 chemokine receptor. Its gene is located on human chromosome 16 along with CCL17 and CCL22. CX3CL1 plays a role in the recruitment of cytotoxic cells and in the elimination of cells that may undergo malignant transformation [15,16,17,18,19,20,21,22,23].

## 2. Material and Methods

PubMed was searched for articles concerning chemokines and CCL in cervical cancer as well as immune checkpoint in cervical cancer on the 26 July 2021. A total of 97 publications resulted from that search. Publications that met the inclusion criteria were included.

## 3. CC Chemokine Ligands (CCL) in Cervical Cancer Biology

### 3.1. CCL2

Monocyte chemoattractant protein-1 (MCP-1) is also called CCL2 and is one of the most important chemokines that regulates the migration and infiltration of monocytes or macrophages. Both CCL2 and its receptor CCR2 have already been described several times in various malignant diseases and there are also corresponding citations for cervical cancer. Initial indications that this chemokine plays a central role in intercellular communication by inducing an intracellular signaling pathway that negatively affects viral transcription in HPV-positive but still benign cervical cells come from the zur Hausen group [24].

Ongoing work on this topic has indicated that CCL2 (MCP-1) is an important factor that is involved in the interaction between mononuclear cells and human papillomavirus (HPV) -infected cervical epithelial cells. CCL2 is also involved in the negative feed-back loop between the expression of the HPV oncogenes E6/E7 and the MCP-1 gene [25]. In the following studies, the same group showed that hyperplastic squamous epithelium, in addition to cervical cancer, has high CCL2 expression and an increased number of infiltrating macrophages [26]. The induction of HPV-induced tumors is driven by the activation of the virus-specific oncoproteins E6 and E7. The E6 protein, which is specific for HPV types 16 and 18, interacts with the E3 ubiquitin protein ligase, resulting in ubiquitination and proteolysis of the tumor protein p53. E7 inactivates the retinoblastoma protein (Rb) through phosphorylation, which leads to an increase in the free eukaryotic transcription factor E2F (E2F) in the HPV-infected cell. This leads to an increase in the cyclin-dependent kinase inhibitor p16, which is also used as an immunohistochemical marker for HPV-associated cancer. Unfortunately, p16 in carcinogenesis is increased by the E7 oncoprotein [27]. Therefore, mRNA in situ hybridization of E6/E7 or the direct detection of the E6/E7 proteins by immunohistochemistry are the preferred methods for the detection of HPV-induced cancer formation [27]. Work that was done by our own group showed that significantly increased E6 staining is associated with advanced T-status and an increased International Federation of Gynecology and Obstetrics (FIGO) classification in cervical cancer [28]. E6, p53, and p16 demonstrated significantly different expression levels in squamous epithelial tissue compared with adenocarcinomas. MDM2 and gal-3 showed positively correlated expression levels in cervical cancer. In cases with negative p16 expression, Gal-3 expression was also correlated with a poor prognosis. A negative correlation was found between the expression of a mutated form of p53 and of E6 in cervical cancer [28]. In addition, E6 and E7 suppressed CCL2 expression in the primary epithelial cells that were derived from the female genital tract in vitro. Other chemokines, including IP-10, IL-8, and CCL5, were less affected [29]. Another in vitro study showed that overexpression of CCL2 in cervical cancer ME180 cells using viral vectors did not affect their proliferation. However, when treated with a low dose of cisplatin, tumor formation was apparently reduced in clones transduced with CCL2. The histological examination revealed that a considerable number of macrophages infiltrated the tumor sites of MCP-1-transduced cells, which gave initial indications of an immune checkpoint involvement of this cytokine [30]. Another genomic approach revealed that the loss of heterozygosity (LOH) at 17q11.2 increased the cumulative relapse-free survival and cumulative overall survival of cervical cancer patients that were lacking tumor cell-associated CCL2 mRNA, suggesting that the tumor-associated macrophages support tumor progression [31].

A histopathologic study showed that the expression of CCL2 and CCL19 were inversely associated with the expression of atypical chemokine receptors (ACRs), including CCX-CKR, DARC, and D6, which have been reported to be involved in cancer invasion and metastasis in patients with cervical squamous cell carcinoma [32]. D6 expression and co-expression with ACR were negatively related to tumor size and recurrence. Furthermore, the CCX-CKR expression is a positive indicator for overall survival [32]. D6 expression was an independent predictor of both overall and recurrence-free survival [32]. In addition, a recent co-expression network analysis of the atypical chemokine receptor 1 (ACKR1) showed an ACKR1 was negatively correlated with lymph node metastasis and prognosis in cervical cancer [33].

Pahne-Zeppenfield et al. have shown in recent work that squamous cell cervical cancer cells activate monocytes to produce their own CCL2 for further monocyte recruitment and to reprogram their function during differentiation and maturation into dendritic cells (DCs) [34]. The study data also showed that cervical cancer cells suppressed the induction of the chemokine receptor CCR7 in phenotypically mature DCs and prevented their migration to a chemokine that resides in the lymph nodes, which is necessary for initiating adaptive immune responses [34].

An in vitro study showed that TPCA-1, a substance also known as an inhibitor of NF-κB, significantly inhibited the release of CCL-2 from HeLa cells (adenocarcinoma of the cervix). TPCA-1 clearly reversed the promoting effect of IL-1*β* on the vitality of HeLa cells. IL-1*β* increased cell migration, proliferation, and invasion of HeLa cells by targeting the NF-κB/CCL-2 pathway. The interaction of IL-1*β*, NF-κB, and CCL-2 could be a promising subject of investigation in the treatment and prevention of adenocarcinoma of the cervix [35].

Interestingly, the CCL2 receptor, CCR2, prevented the progression from squamous intraepithelial lesions to invasive cervical carcinoma if carrying the polymorphism CCR2-64I [36]. Additional work of the same group showed that CCR2-64I polymorphism might contribute to the establishment of high-grade squamous intraepithelial lesions through the disruption of the naturally fragile immune response that is directed towards human papillomavirus infection [37]. Another team showed that the CCR2-64I variant was associated with a decreased risk of cervical cancer; homozygote carriers of the 64I variant had an odds ratio of 0.31 (0.12–0.77). This association has been demonstrated in both carriers and non-carriers of the HLA DQB1 * 0602 cervical cancer risk allele [38].

### 3.2. CCL3, CCL4, CCL5, and CCL8

The chemokine CCL5, better known as RANTES, also belongs to the family of CC chemokines. It is secreted by T lymphocytes after activation and by fibroblasts, epithelial cells, and endothelial cells after stimulation with TNF-*α*, IL-1*β*, and IFN-*γ*. CCL5 is a powerful chemoattractant for monocytes, memory T cells, basophils, and eosinophils. In cervical cancer, the CCL5 content was significantly increased in the primary tumor and also in metastatic lesions (lymph nodes or skin) [39].

CCR5 belongs to the group of G protein-coupled receptors, which act as chemokine receptors in the CC chemokine family. CCR5 binds several chemokines: CCL3, CCL4, CCL5, and CCL8. Chemokine receptor gene polymorphism CCR5 Delta32 patients showed a significant risk enhancement in stage IB cervical cancer [40].

In connection with CCL8 it has already been shown that ZEB1, induced by hypoxia, promotes the progression of cervical cancer through CCL8-dependent tumor-associated macrophage recruitment [41]. In particular, ZEB1 that was induced by hypoxia activated the expression of CCL8, which in turn led to macrophages being attracted via the CCR2-NF-κB signaling pathway [41]. In addition, it was found that, based on the data analysis of the Cancer Genome Atlas (TCGA), ZEB1 and CCL8 are independent prognostic factors in cervical cancer patients [41].

### 3.3. CCL17 and CCL19

A newly investigated chemokine is CCL17. In vitro studies on HeLa and SiHa (squamous cell carcinoma) cells showed that hypoxia increased the expression of the CCL17 receptor (CCR4) [8]. Recombinant CCL17 led to a dose-dependent increase in cell proliferation in these cell cultures [8]. The blockade of CCL17 with the help of anti-human CCL17 antibodies (*α*-CCL17) induced the opposite effect [8]. The proliferation of HeLa and SiHa cells could be induced in vitro with the help of hypoxia; treatment with *α*-CCL17 reversed this effect [8]. The proliferation of HeLa and SiHa cells could be reduced by stimulation with the inhibitor for *c*-Jun *N*-terminal kinase (JNK) or a signal transducer and activator of the transcription 5 signaling pathway (STAT5) [8]. These results show that an increased CCL17 expression in cervical lesions is a significant inducer of the proliferation of squamous cells and adenocarcinoma of the cervix via the JNK and STAT5 pathways [8].

Another member of C-C chemokine receptor type is CCR7. CCR7 is a protein that, in humans, binds the chemokines ligand 19 (CCL19/ELC) and (C-C motif) ligand 21 (CCL21). Studies on this receptor showed that HPV16-E6 reactive T cells are preferred in the CD45RA + CCR7 + T cell subpopulation of tumor infiltrating lymphocytes, peripheral blood lymphocytes, and T cells that are harvested from the draining of lymph nodes (T-LN) in patients with squamous cell carcinoma of the uterine cervix. This, in turn, indicates that successful immune defense against HPV16 + tumor cells is inhibited in those patients. The unique CD45RA +/CCR7 + phenotype of HPV antigen-induced T cells can serve as a specific marker for dysfunctional T cells in HPV16-induced squamous cell cervical cancer [42].

### 3.4. CCL20 and CCL22

CCL20 is a chemokine that acts as a chemotactic factor for lymphocytes and neutrophils, but not monocytes. CCL20 is involved in the recruitment of IL17 positive T helper 17 cells (Th17) and the association of T regulatory cells (Treg) at the foci of inflammation. During cervical carcinogenesis, HPV16 E6/E7 induces downregulation of CCL20. The reduced ability of the immune system to control an HPV infection can be explained by this inhibition of CCL20 [43]. In addition, CCL20 is one of its target genes of MicroRNA 21 (miR-21) [44]. The same group showed that miR-21 is significantly overexpressed in human cervical squamous cancer tissues and cell lines [44]. The measured amount of miR-21 correlates with the tumor differentiation and the nodal status. It is also known that miR-21 regulates the proliferation, apoptosis, and migration of HPV16-positive cervical squamous cells [44].

Th17 cells accumulate within tumor tissues and correlate with the recurrence of cervical cancer patients [11]. Recent in vitro migration assays that were established by Yu et al. showed that CCL20 had effective chemotaxis to circulating Th17 cells. In summary, it can be said that Th17 cells are preferentially recruited into tumor tissue via the CCR6-CCL20 signal transduction pathway. This newly described pathway could serve as a new therapeutic target for cervical cancer [11]. Another study showed that stromal fibroblasts induce CCL20 through IL6/C/EBP*β* to support the recruitment of Th17 cells during cervical cancer progression [10]. With the help of this study, a novel molecular mechanism was defined, which explains how cervical neoplastic cells form their local microenvironment. This is done by stimulating fibroblasts to support the infiltration of Th17 cells in a paracrine IL-6/C/EBP*β*-dependent manner [10].

The CCL20/CCR6-induced epithelial-mesenchymal transition (EMT) development via both Erk1/2 and Akt signaling pathway in squamous cell cervical cancer leads to progression/metastasis [45]. Astrocyte Elevated Gene-1 (AEG-1) has been described as an important mediator involved in EMT [45]. AEG-1 proteins are highly expressed in squamous cell cervical cancer tissues and closely correlated with FIGO stage and metastasis [45].

The chemokine CCL20 could be inhibited via E6 and E7 in lesions of the high-risk HPV type to evade the immune response. This was assumed by a study by Jiang and Xue, that showed that there exists a correlation of E6 and E7 levels in high-risk HPV16 type cervical lesions with CCL20 and Langerhans cells [46].

The chemokine CCL22 is known to recruit Treg into tumor tissue and is also expressed in many human tumors [5]. However, the prognostic role in cervical cancer for CCL22 was not clarified until recently [5]. Within a study of our group, we retrospectively analyzed the clinical significance of the expression of CCL22 and FOXP3 in 230 cervical cancer patients. With the help of a tissue microarray (TMA), immunohistochemical staining analyses of CCL22 and FOXP3 were carried out [5]. The number of infiltrating CCL22^hi^ cells were also positively correlated with the number of infiltrating FOXP3+ cells [5]. The CCL22^hi^ group had a lower overall survival (OS) compared to the CCL22^lo^ group [5]. In addition, CCL22^hi^ is an independent prognostic factor of shorter OS [5]. In the combination group, CCL22h^hi^FOXP3^hi^, the overall survival was significantly lower than the overall survival in the combination group, CCL22^lo^FOXP3^lo^, regardless of the FIGO stage and disease subtype [5]. Cox regression analysis showed that for CCL22^hi^FOXP3^hi^ this combination was an independent indicator of a shorter OS. In the group CCL22^hi^FOXP3^hi^, although PFS was in cervical adenocarcinoma significantly lower than that of the group CCL22^lo^FOXP3^lo^, CCL22^hi^FOXP3^hi^ was not an independent indicator [5]. CCL22 was mainly expressed in M2-like macrophages in CC and induced by cervical cancer cells. The results of our study show that cervical cancer patients with elevated CCL22 + infiltrating cells need more aggressive treatment [5].

**Figure 1:**
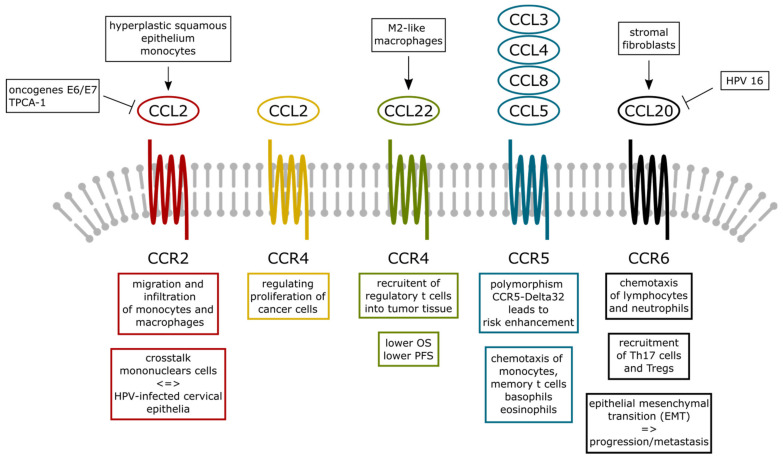
Summary of the described chemokines of the CC chemokine proteins (or *β* chemokine proteins) family. These chemokines have two adjacent cysteine (amino acids) near their amino terminus. Although at least 27 different member of this subgroup have been reported for mammals, referred to as CC chemokine ligands (CCL)-1 to -28, we focused in this review on 10 CCL chemokines and described ligand receptor interaction for CCL2, -3, -4, -5, -8, -20 and -22 specifically for their function in cervical cancer. Abbreviations: HPV—human papilloma virus; CCL—C-C Motif Chemokine Ligand; C-C chemokine receptor; E6—HPV E6 protein; E7—HPV E7 protein; OS—overall survival rate; TPCA-1-2-[(aminocarbonyl)amino]-5-(4-fluorophenyl)-3-thiophenecarboxamide; Treg—T regulatory cells.

A summary of the C-C chemokines, its receptors and main function in cervical cancer is presented in Table 1. The interaction between C-C chemokines and its receptors is displayed in Figure 1.

## 4. CXC Chemokines and Its Receptors in Cervical Cancer

### 4.1. CXCL1, CXCL2, and CXCL3

CXCL1 belongs to the family of CXC chemokines, which act as a chemoattractant for several immune cells, particularly neutrophils. It also plays an important role in regulating the immune and inflammatory responses. CXCL1 was previously named GRO1 oncogene, GRO*α*, Neutrophil Activating Protein 3 (NAP-3), and Melanoma Growth Stimulating Activity-Alpha (MGSA-*α*) [48]. Interestingly, it is seminal plasma that acts via angiogenic chemokine expression in HeLa cells and thus regulates vascular function specifically via CXCL1 signal transduction [49]. In vitro experiments showed that HeLa cells induced the formation of endothelial cells into a network of tubular structures via the CXCR2 receptor on HUVECs. The data from this study reveal a molecular mechanism by which seminal plasma can modulate the vascular function in HeLa cells with the help of IL-8 and the pro-angiogenic chemokine CXCL1 [49].

A nanomedical study showed that synthesized Annona muricata silver nanoparticles exhibited potent anticancer activities against cervical and prostate adenocarcinomas through the regulation of CASP9 and the CXCL1/CXCR2 gene axis [50].

AKIP1 in cervical cancer cells stimulates the expression of CXCL1, CXCL2, and CXCL8 [51]. By binding to the endothelial receptor CXCR2, these chemokines are involved in endothelial tube formation as well as in the proliferation of cervical cancer cells and in the clone-formation that is induced by the overexpression of AKIP1 [51]. AKIP1-induced chemokine expression was inhibited by a negative inducer of the nuclear factor kappa B kinase subunit *β* [51]. Therefore, AKIP1 is critical to the angiogenesis and growth of cervical cancer by increasing levels of the NF-κB-dependent chemokines CXCL1, CXCL2, and CXCL8 [51].

CXCL3 expression was analysed in a combined in vivo and in vitro study and strongly correlated with CXCL5 expression in HeLa cells [52]. In vitro, HeLa cells overexpressing CXCL3 showed enhanced proliferation and migration activities [52]. The overexpression of CXCL3 was also examined in a HeLa cell tumor xenograft model [52]. Mechanistic studies showed that CXCL3 overexpression affected the expression of genes that were associated with the extracellular signal-regulated kinase (ERK) pathway. These studies included ERK1/2, Bcl-2, and Bax. However, exogenous administration of the ERK1/2 blocker PD98059 weakened the CXCL3-induced proliferation and migration effects [52].

### 4.2. CXCL5, CXCL6, CXCL8, and CXCL10

CXCL5 and its receptor CXCR2 are expressed by HeLa cells and CXCL5 is upregulated in cervical cancer tissues [53]. The expression of CXCL5 correlates positively with age, but not with clinical stages and tumor infiltration [53]. The overexpression of CXCL5 and the exogenous administration of CXCL5 contributed to the proliferation and migration activity of Hela cells in vitro. In addition, overexpression of CXCL5 also promoted the growth of HeLa cells in a nude mouse xenograft model [53]. CXCL5 overexpression also regulated the expression of tumor-related genes such as ERK, *p*-ERK, AKT, *p*-AKT, DIABOL, NUMB, NDRG3, and CXCR2 at the gene level [53]. In addition, CXCL5 contributes to the tumorigenicity of cervical cancer and is post-transcriptionally regulated by miR-577 [54]. Knockdown of CXCL5 with specific siRNA transfection in Hela and SiHa cells significantly inhibited cell proliferation and migration and induced apoptosis in vitro [54]. The authors also showed that CXCL5 was a direct target of miR-577 [54].

By inhibiting CXCL6, MicroRNA-101-5p inhibits the growth and metastasis of cervical cancer cells [55]. CXCL6 is the target protein of miR-101-5p in cervical cancer and over-regulation of miR-101-5p reduced the tumor growth of cervical cancer cell in vivo [55]. Finally, the mRNA level of CXCL6 was negatively associated with the miR-101-5p level in cervical cancer tissue [55].

CXCL8, a newly investigated cytokine is highly expressed in HeLa and Caski (squamous cell carcinoma of the cervix) cell lines compared with normal cervical tissues in microarray datasets (GSE9750 and GSE7803) [56]. In cervical cancer tissues and cell lines, CXCL8 mRNA and protein expression were increased compared to normal cervical tissues and cervical epithelial cell lines [56]. CXCL8 protein expression was significantly correlated with the clinical stage, distant metastasis, histological type, and histological grade [56]. In summary, it can be said that the high expression of CXCL8 is a negative independent prognostic parameter for cervical cancer patients [56]. In addition, miR-302c-3p and miR-520a-3p suppressed the proliferation of cervical carcinoma cells by targeting CXCL8 [57]. The inhibition of CXCL8 in combination with miR-302c-3p and/or miR-520a-3p overexpression had proliferation-suppressing and apoptosis-stimulating effects on cervical cancer cells. In contrast, the restoration of CXCL8 weakened the miR-302c-3p and miR-520a-3p-induced anti-proliferative and pro-apoptotic effects [57].

CXCL10, also known as interferon-gamma-induced protein 10 (IP-10) or small-inducible cytokine B10, decreased as the disease progressed. Accordingly, the prognosis of patients with a low CXCL10 expression in cervical cancer was poor. In addition, CXCL10 levels were significantly inversely correlated with vascular endothelial growth factor (VEGF) levels in cervical cancer. CXCL10 could work to suppress VEGF-associated angiogenesis in the future and can be recognized as a prognostic indicator for both squamous cell and adenocarcinoma of the uterine cervix [58]. Additional experimental data showed that CXCL10 can inhibit the growth of cervical carcinoma through modulating the formation of micro-vessels and the expression of HPV oncoproteins E6 and E7. The results of this group also provide further evidence of the anti-tumor effects of CXCL10, which may be relevant in further exploration of the potential applications of this molecule in the treatment of cervical cancer [59]. Recently, a group found that CXCL10 enhances radiotherapy effects in HeLa cells through cell cycle redistribution [60]. Flow cytometry showed that the overexpression of CXCL10 in HeLa cells led to a prolonged G1 phase and a shortened S phase 72 h after transfection. The subsequent Western blot analysis showed that p27 (Kip1) was upregulated in CXCL10-treated HeLa cells and cyclin E was downregulated [60]. Based on these in vitro findings, mouse models of adenocarcinoma of the cervix were created by inoculation with HeLa cells and were treated by combining intravenously administered plasmid-encoding CXCL10 with direct radiation. The results showed a significant increase in tumor growth inhibition, reduced vessel density, decreased cell proliferation, and increased apoptosis in the cervical cancer cells of the combination therapy group. Therefore, CXCL10 gene therapy in combination with radiation therapy could be a novel and effective therapeutic strategy for the treatment of squamous cell cervical cancer [17].

### 4.3. CXCL11 and CXCL12

The chemokine ligands CXCL11 (which also activates CXCR3) and CXCL12 (which also activates CXCR4) bind to the chemokine receptor CXCR7. CXCR7 does not activate the G protein signaling but does activate *β*-arrestin [61]. Interestingly, the CXCR7 expression is associated with reduced disease-free and disease-specific survival in cervical cancer patients [62]. A recent study produced similar results, showing that the expression of CXCR7 and EGFR was associated with shorter disease-free survival (DFS) and overall survival (OS) [63]. The multivariate analyses indicated that CXCR7 was independently associated with DFS and OS [63]. The prevalence of recurrences and distant metastases was significantly lower in the group with external pelvic irradiation with brachytherapy than in the group with radical hysterectomy during CXCR7 expression. In addition, the CXCR7 knockdown significantly reduced the proliferation and invasion of squamous cell cervical cancer cells in vitro [63]. Furthermore, CXCL12 is highly expressed in HeLa cell lines [64]. The silencing of CXCR7 or CCX733 treatment as opposed to CXCR4 silencing or AMD3100 treatment suppressed the proliferation, migration, and invasion of HeLa cells that were stimulated by CXCL12 [64]. In a xenograft tumor model, CXCR7 silencing or CCX733 treatment inhibited the volumes and weight of xenograft tumors. In addition, the inhibition of CXCR7 decreased the expression level of MMP2 and MMP9, and in contrast, CXCR7 inhibition increased TIMP-1 and TIMP-2 in vivo [64].

Specific for CXCL12 is the CXCR-4 receptor, an alpha chemokine receptor with strong chemotactic activity for lymphocytes. CXCL12 is the major modulator of trafficking of myeloid cells (precursors, MDSC, and TAMs). A histopathological study showed that CXCR4 expression is significantly higher in older patients than in younger patients. This increased CXCR4 expression has also been demonstrated in patients with squamous cell and adenocarcinoma of the cervix who had a large tumor size, deep stromal invasion, involvement of the lymphatic space, or lymph node metastasis [65]. Although the chemokine receptor CXCR4 has been shown to be expressed by most cancers, it was originally reported that CXCR4 regulates the migration of lymphocytes into the inflammatory tissues [65]. In vitro experiments were carried out to further evaluate the effect of CXCL12 on the proliferation of cervical cancer cells. Hela cells were cultured for 72 h and exposed to CXCL12 with and without CXCR4 monoclonal antibody (mAb). The authors found that CXCR4 was expressed on SCC cells in all cervical cancer tissues, metastatic lymph node, and Hela cells, but not in normal cervical tissue. CXCL12 is also expressed on immune cells in lymph nodes. CXCL12 induced the directed migration of Hela cells in a concentration-dependent manner; this migration induction could be inhibited by CXCR4 mAb [66]. An additional study showed that CXCR4 expression is associated with pelvic lymph node metastasis in cervical adenocarcinoma and the addition of CXCL12 provoked significant signal transduction events, including chemotaxis and rescue from apoptosis. The effects described were most likely mediated by the activation and phosphorylation of the extracellular signal-regulated kinase 1/2 and AKT pathways [67]. Another approach to studying CXCL12 and its receptor CXCR4 took advantage of the fact that CXCR4 is the most commonly expressed chemokine receptor in most cancers and has been linked to tumor spread and poor prognosis. For this reason, several CXCR4 antagonists have already been tested as potential anti-tumor agents. A new approach to the discovery of chemokine receptor antagonists is the use of bacteria, as described by Walenkamp et al. [68]. Bacteria produce chemokine receptor inhibitors to escape clearance by innate immune cells [68]. The superantigen-like 10 produced by staphylococci inhibited CXCL12-induced calcium mobilization and cell migration in HeLa cells [68]. The group Amin et al. found that the invasion of E6/E7-positive cancer cell lines (HeLa and TC-1) in Matrigel is stimulated by CXCL12-CXCR4 interaction and subsequent Rho/ROCK activation [69]. A high proportion of the cells that were expressing membrane-associated CXCR4 were found in pulmonary metastatic foci that were generated by TC-1 cells. Cell adhesion and invasion can be abolished by the immunological blockade of CXCR4, as in vitro and in vivo models have shown. These findings can help explain the CXCL12/CXCR4-controlled metastasis process in cervical cancer [69]. Inhibition of CXCR4 or Hedgehog (Hh) gene activity during tumor growth under hypoxic conditions reduced the size of the primary tumor and additionally reduced lymphatic metastasis to levels that were below those seen in control mice that were exposed to normoxic conditions [70].

The invasion of HeLa cells that are induced by CXCL12 is determined by the action of matrix metalloproteinase-9 (MMP-9), as Brule et al. showed [71]. The CXCL12-mediated cell invasion could be inhibited by preincubation of HeLa cells with heparin or with anti-heparan sulfate antibodies or with beta-D-xyloside. In addition, the downregulation of Syndecan-4, a heparan sulfate proteoglycan, decreased CXCL12-mediated HeLa cell invasion. Glycosaminoglycans on Syndecan-4 are likely involved in CXCL12-mediated cell chemotaxis [71]. Jaafar et al. investigated the correlation between CXCL12 expression and FoxP3 + cell infiltration in human papillomavirus infection and also in the clinicopathological progression of cervical cancer [72]. FoxP3 and CXCL12 expression correlated significantly in patients with squamous and glandular neoplasia [72]. Another approach that was proposed by Cai et al., took advantage of the fact that SIVmac_239_-Nef downregulates the cell surface expression of CXCR4 in tumor cells and, therefore, proliferation, migration, and angiogenesis are inhibited [73]. In further experiments, a downregulation of the cell surface of CXCR4 in HeLa cells was observed after the Nef transfection. The proliferation as well as the migration of Nef-transfected HeLa cells in vitro was significantly reduced. The in vitro tube formation was also significantly lower after Nef transfection and CXCR4 knockdown with siRNA [73]. Another experimental study showed that oligomannurarate sulfate inhibits CXCL12-mediated proliferation and invasion of HeLa cells in vitro [74].

CXCL12 induces directed cell migration and also spontaneous metastasis, presumably via activated mTOR (a mechanistic target of rapamycin) in a very effective and pertussis-sensitive manner. Further experiments showed that the inhibition of the mTOR complex 1 (mTORC1) by rapamycin and mTORC1/mTORC2 by Torin2 reduced the directed cell migration in the direction of CXCL12. The same effects could also be observed through knock-down key components of mTORC1/2, Raptor, and Rictor [75]. Based on that finding, mTORC1 could be a suitable therapeutic target in the treatment of human malignancies as melanoma cells use CXCR4 for their metastatic spread [75].

In cervical cancer cell lines and primary tumor biopsies, CXCL12 is often downregulated and its promoter is hypermethylated [15]. Exogenous treatment with recombinant CXCL12 inhibited metastasis-promoting cell migration, cell invasion, and anchorage-independent cell growth events in cervical cancer cell lines (HeLa, SiHa and C-33A) [15].

The CXCL12/CXCR4 chemokine pathway is expressed in cervical cancer. The CXCL12/CXCR4 signaling pathway plays an important role in the development of cervical cancer, the further course of this malignant disease, the development of metastases, and the response to radiation therapy. Preclinical studies with fractionated standard radiation therapy and simultaneous weekly administration of cisplatin plus the CXCR4 inhibitor Plerixafor (AMD3100) in xenografts from patients with orthotopic cervical cancer showed an improved response of the primary tumor and a reduced number of lymph node metastases without an increase in the early- or late-stage of side effects [76].

An in vitro study with Hela cells showed that inhibition of CXCL12 decreased cell viability and increased cellular apoptosis in radiation-treated cells [77]. After treatment with CXCL12 siRNA in the same cell culture model, the expression level of CD44 was downregulated and the expression level of CXCR4 was upregulated [77]. The regulation effect that is described above also occurred during irradiation [77]. Another in vitro study used plerixafor in combination to radio/chemotherapy and the combined treatment-induced increases in the CXCL12/CXCR4 signaling [78]. The authors showed that this combination improves primary tumor response and reduces intestinal side effects, and that this combination warrants testing in future clinical trials [76].

In contrast to former studies, a more recent study shows that the chemokine CXCL12 and its receptor CXCR4 are constitutively overexpressed in human cancers, especially cervical cancer [79]. The interaction of CXCL12 with CXCR4 and the subsequent signal transduction plays an important role in tumor progression and metastasis. This interaction also appears to be important in the therapy-induced recruitment of CXCR4-expressing cytotoxic immune cells [79]. In a recent study, Hartimath et al. investigated the feasibility of *N*-[11C] methyl-AMD3465 positron emission tomography (PET) to monitor the changes in CXCR4 density in tumors after single-fraction local radiotherapy or in combination with immunization [79]. The authors demonstrated the feasibility of *N*-[11C] -methyl-AMD3465 PET imaging to monitor treatment-induced changes in the density of CXCR4 receptors in tumors. Further evaluation of CXCR4 as a potential imaging biomarker could be warranted for the development of broader anti-tumor therapies [79].

### 4.4. CXCL13 and CXCL16

Recently it was shown that hypermethylation of single CpG dinucleotides at the promoter of CXCL13 gene stimulates cell migration in cervical cancer [80]. The downregulation of CXCL13 has been linked to hypermethylation of certain genes in cervical cancer cell lines and primary tumor biopsies [80]. For example, a CpG dinucleotide on the HIF-1a transcription factor motifs in the promoter element of CXCL13 in cervical cancer cells was consistently methylated and thus associated with HIF-1a [80]. In a xenograft model with CXCL13 overexpression and S110 treatment, tumor growth and liver metastasis were suppressed, whereas its low expression increased the risk of death in cervical cancer patients [80].

A new cytokine (CXCL16) was investigated together with the chemokine receptor combination CXCL12/CXCR4 and CXCL16/CXCR6 in cervical intraepithelial neoplasia (CIN) and cervical cancer [81]. Huang et al. were able to show that an increased level of co-expression of CXCL12/CXCR4 and CXCL16/CXCR6 in CIN and cervical cancer indicates a persistent process in the development of cervical cancer. Moreover, the CXCL16/CXCR6 complex may be useful as a biomarker and a valuable prognostic factor for cervical cancer [81].

**Figure 2:**
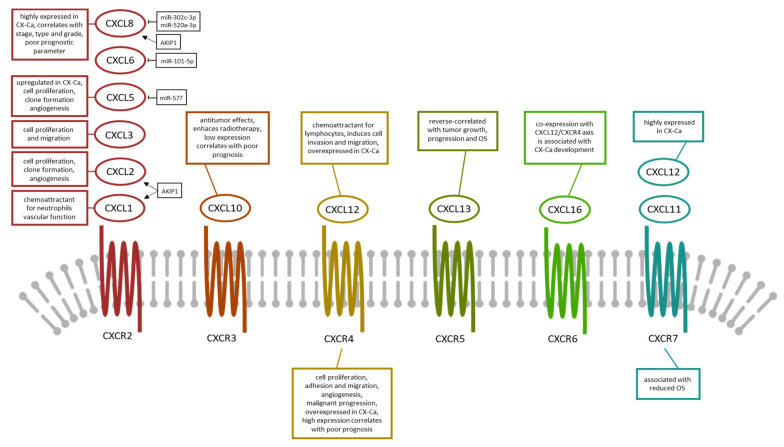
Summary of the CXC-chemokines, its receptor binding partner and main function in cervical cancer. A total of six CXC-chemokines (CXCL-1, -2, -3, -5, -6, *&* -8 bind to the same receptor CXCR2. Abbreviations: CX-Ca—carcinoma of the uterine cervix; CXCL—C-X-C motif chemokine; CXCR—C-X-C motif receptor; AKIP1—A-Kinase Interacting Protein 1; OS—overall survival.

A summary of the C-X-C chemokines, its receptors and main function in cervical cancer is presented in Table 2. The interaction between C-X-C chemokines and its receptors is displayed in Figure 2.

## 5. The Role of Chemokines in Checkpoint Activation/Inhibition in Cervical Cancer

Checkpoint inhibitors are among the most promising therapeutic approaches for the treatment of a variety of cancers such as cervical cancer, leading to strong immune responses against tumor cells by blocking PD1/PD-L1 or TIM-3/Gal-9.

In a recent study, Moeini et al. investigated whether the therapeutic effect of a DNA vaccine, which codes for the human papillomavirus type 16 (HPV-16) E7, through the combined application of an immune checkpoint blockade against the programmed death-1 (PD-1) signaling pathway and secondary lymphoid tissue chemokine (SLC), also known as the CCL21 adjuvant, can be enhanced in a mouse cervical carcinoma model [83]. As a result, the authors were able to show that vaccination with the described DNA vaccine in combination with the CCL21 adjuvant plus PD-1 blockade greatly increased cytotoxic T lymphocyte production and the lymphocyte proliferation rate and strongly inhibited tumor progression. The vaccine in combination with adjuvant and blockade also significantly reduced intertumoral VEGF, IL-10, and spleen IL-4, but induced the expression of spleen IFN-*γ* [83].

Although the inhibition of the PD-L1/PD-1 immune checkpoints is one of the most promising approaches in immunotherapy, it is only successful in subpopulations of patients. Recent work has found recurrent copy number gains (CNG) on chromosome 9p involving PD-L1 in a number of cancers, including cervical cancer. A list of 75 genes using the TCGA dataset was recently identified. This list included genes that were severely upregulated in tumors with chromosome 9p gains including the chemokines CCL4, CCL8, CXCL10, and CXCL11 [84]. These changes were found for many cancers.

Tian et al. showed that CCR7 has the potential to be a prognosis marker for squamous cell cervical carcinoma cells and an index for tumor microenvironment change [85]. The CIBERSORT analysis showed a positive correlation between plasma cells, CD8 + T cells, and regulatory T cells and CCR7 expression within this study, suggesting that CCR7 could play a crucial role in maintaining the immunological dominance status for the tumor microenvironment [85].

**Figure 3:**
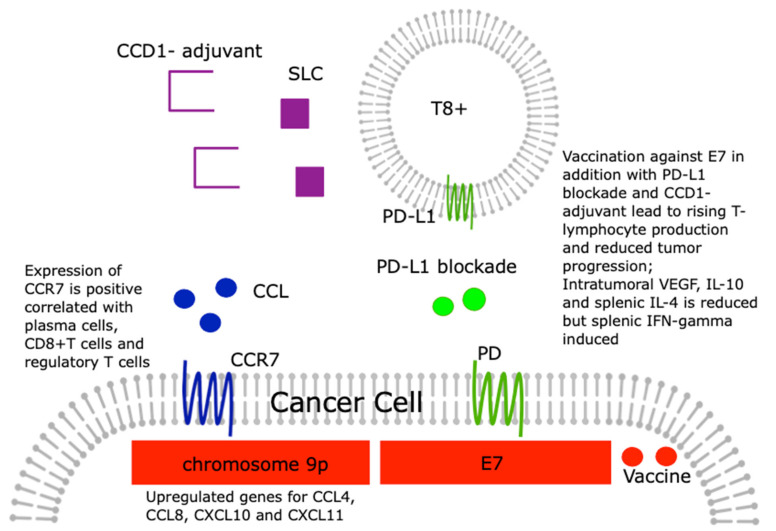
Systematic depiction of the booster E7 vaccination effect by simultaneous application of PD-L1 and SLC, and correlation between plasma cells, CD8+ T cells, and regulatory T cells and the CCR7 expression. Abbreviations: CCR—C-C motif receptor; CCL—C-C motif ligand; CXCL—C-X-C motif ligand; PD-L1 - programmed cell Death protein 1 ligand; E7—HPV E7 protein; IL—interleukin; VEGF—vascular endothelial growth factor; IFN—interferon; T8+—CD8+ T cells; SLC—secondary lymphoid tissue chemokine.

These coherences are visualized in Figure 3.

## 6. Conclusions

Cervical cancer is the fourth most common cancer worldwide and the fourth leading cause of cancer death in women worldwide. About half of patients with cervical cancer have locally advanced disease for whom surgery is not an option. However, these cases would be potentially curable with the use of radiotherapy and cisplatin chemotherapy. Unfortunately, some tumors are resistant to treatment. Lymph node infiltration and recurrences are major problems in patients with advanced disease. New targeted therapies that can overcome treatment resistance and reduce metastases are urgently needed. Therefore immune-oncological research with a specific focus on chemokines and their receptors may be promising. To summarize the 27 different members of this subgroup that have been reported for mammals, referred to as CC chemokine ligands (CCL)-1 to -28, CCL-2 is the most investigated chemokine within this group of chemokines. Interestingly, macrophages/dendritic cells also seem to produce their own CCL2 in the cervical cancer environment, and, therefore, this chemokine together with others like CCL5 may be responsible for attracting tumor associated macrophages (TAM), their infiltration, and inducing their polarization toward cancer-promoting M2-phenotype [86]. The dominant member of the CXC chemokine subfamily concerning cervical cancer is CXCL12 resulting in 52 PubMed hits. Interestingly, the estrogen receptor α promotes cancer cell invasion via the increase of and cross-talk with infiltrated macrophages through the CCL2/CCR2/MMP9 and CXCL12/CXCR4 signaling pathways at least in lung cancer [87]. To conclude, chemokines play a direct role in cervical cancer biology. On the other hand, chemokines induce TAM infiltration and checkpoint activation. This is an open field of research especially considering cervical cancer.

## Figures and Tables

**Table 1:** Summary of the C-C-chemokines, its distribution, and the main function in cervical cancer.

Chemokine.	Effects of the Chemokine	Effects in Cervical Cancer	Receptor	References
CCL2 (MCP-1)	Migration, infiltration of monocytes and macrophages	Crosstalk between mononuclear cells and HPV-infected epithelia	CCR-2	[24,26]
CCL5 (RANTES)	Chemoattractant for monocytes, memory T-cells, basophils, eosinophils	Elevated in primary tumor and metastatic lesions	CCR-5	[39]
CCL8 (MCP-2)	Recruitment of macrophages via CCR-2- NFκB-pathway	Independent prognosticator for cervical cancer progression	CCR-2	[41]
CCL17 (TARC)	Dose-dependent cell proliferation	High level regulates proliferation of cervical cancer cells via JNK/STAT5 pathways	CCR-4	[8]
CCL19 (MIP-3*ß*), CCL21	Proinflammatory	Dysfunctional T-cells in HPV16 positive cervical cancer cells	CCR-7	[42,47]
CCL20 (MIP-3A)	Chemoattractant for lymphocytes and neutrophils, recruitment of Th17 and Treg cells	Down-regulation by HPV16 E6/E7	CCR-6	[11]
CCL22	Recruitment of Treg cells	Independent predictor for shorter OS	CCR-4	[5]

Abbreviations: HPV—human papilloma virus; NFκB—nuclear factor ‘kappa-light-chain-enhancer’ of activated B-cells; JNK—c-Jun N-terminal kinases; STAT5—Signal transducer and activator of transcription 5; HPV16—HPV type 16; E6—HPV E6 protein; E7—HPV E7 protein; OS—overall survival rate.

**Table 2:** Summary of the CXC-chemokines, its distribution, and main function in cervical cancer.

Chemokine (Alternate Names)	Effects of the Chemokine	Effects in Cervical Cancer	Receptor	References
CXCL1 (GRO1 oncogene, GRO*α*, NAP-3, MGSA-*α*)	Chemoattractant for immune cells, endothelial tube formation	Increased cervical cancer angiogenesis (AKIP1-dependent)	CXCR2	[49]
CXCL2 (GRO*β*)	Endothelial tube formation	Increased cervical cancer angiogenesis (AKIP1-dependent) - > increased Cervical cancer cell proliferation	CXCR2	[51]
CXCL3 (GRO*γ*)	Enhanced proliferation and migration activities	Potential tumor marker and interference target	CXCR2	[52]
CXCL5 (ENA78)	Cell proliferation and migration, regulation of expression of tumor-related genes	Contributes to the tumorigenicity of cervical cancer	CXCR2	[53,54]
CXCL6 (GCP2)	Chemotactic for neutrophil granulocytes	Inhibition via Micro-RNA-101-5p leads to Inhibition of tumor growth and metastasis	CXCR1, CXCR2	[55,82]
CXCL8 (IL-8)	Proinflammatory, endothelial tube formation	Increased cervical cancer angiogenesis (AKIP1-dependent), Expression is correlated with clinical stage, distant metastasis, histological type and grade	CXCR1, CXCR2	[56,57]
CXCL10 (IP-10, small-inducible cytokine B10)	Suppression of angiogenesis, modulating formation of micro vessel and expression of E6 and E7	Prognostic indicator for cervical cancer, May be used as gene therapy in combination with radiotherapy	CXCR3	[58,59]
CXCL11 (I-TAC)	Chemotactic for interleukin-activated T-cells	Higher expression of CXCR7 is associated with shorter DFS and OS	CXCR3, CXCR7	[63,82]
CXCL12 (SDF1)	Induction of directed cell migration	Chemotaxis and rescue from apoptosis, linked to tumor dissemination and poor prognosis	CXCR4, CXCR7	[65,66,74]
CXCL13 (BCA1)	Inhibits cell migration	Low expression is associated with risk of death	CXCR3, CXCR5	[80]
CXCL16	Co-expression with CXCL12/CXCR4 - > durative process in cervical cancer development	Biomarker, prognostic factor	CXCR6	[81]

Abbreviations: CXCL—C-X-C motif chemokine; CXCR—C-X-C motif receptor; AKIP1—A-Kinase Interacting Protein 1; OS—overall survival; IL—interleukin; BCA1—B cell-attracting chemokine 1; SDF1—stromal cell-derived factor 1; I-TAC—Interferon-inducible T-cell alpha chemoattractant; IP—interferon-gamma induced protein; ENA78—Epithelial Neutrophil Activating Peptide 78; GRO—growth related oncogene; MGSA *α*—Melanoma Growth Stimulatory Activity *α*; NAP-3—Neutrophil Activating Protein 3.

## Data Availability

All data were obtained from pubmed and they are freely available.
